# Research Progress in Fiber Bragg Grating-Based Ocean Temperature and Depth Sensors

**DOI:** 10.3390/s25010183

**Published:** 2024-12-31

**Authors:** Xinyu Zhao, Chenxi Wei, Lina Zeng, Li Sun, Zaijin Li, Hao Chen, Guojun Liu, Zhongliang Qiao, Yi Qu, Dongxin Xu, Lianhe Li, Lin Li

**Affiliations:** 1 College of Physics and Electronic Engineering, Hainan Normal University, Haikou 571158, China; 202206071348@hainnu.edu.cn (X.Z.); 202206071336@hainnu.edu.cn (C.W.); zenglina@hainnu.edu.cn (L.Z.); lisun_2014@163.com (L.S.); lizaijin@hainnu.edu.cn (Z.L.); 15948713468@163.com (H.C.); 068006@hainnu.edu.cn (G.L.); qzhl060910@hainnu.edu.cn (Z.Q.); quyi@hainnu.edu.cn (Y.Q.); 2 Key Laboratory of Laser Technology and Optoelectronic Functional Materials of Hainan Province, Hainan Normal University, Haikou 571158, China; jilinchangchun@yeah.net; 3 Hainan International Joint Research Center for Semiconductor Lasers, Hainan Normal University, Haikou 571158, China; lilianhehnnu@126.com

**Keywords:** fiber Bragg grating, temperature sensors, pressure sensors, synchronized acquisition of temperature and pressure data

## Abstract

Fiber Bragg gratings (FBGs) are widely used in stress and temperature sensing due to their small size, light weight, high resistance to high temperatures, corrosion, electromagnetic interference, and low cost. In recent years, various structural enhancements and sensitization to FBGs have been explored to improve the performance of ocean temperature and depth sensors, thereby enhancing the accuracy and detection range of ocean temperature and depth data. This paper reviews advancements in temperature, pressure, and dual-parameter enhancement techniques for FBG-based sensors. Additionally, the advantages and disadvantages of each method are compared and analyzed, providing new directions for the application of FBG sensors in marine exploration.

## 1. Introduction

The dynamics of the ocean can be predicted through the analysis of various parameters [1]. Through marine sensing, key parameters can be obtained, which typically include physical, chemical, geographical, and biological factors. Physical parameters primarily include the ocean temperature, salinity, depth, and current. These data not only aid scientists in understanding changes in the oceanic ecosystem but also contribute to the sustainable management of the marine environment. Among these, temperature and pressure are essential for measuring other parameters such as the ocean current, density, and heat content. Real-time, on-site monitoring of these parameters is indispensable in fields such as marine resource development, offshore industries, global warming mitigation, and naval defense. Therefore, temperature and pressure sensors, as fundamental tools in oceanographic measurements, play a crucial role in areas such as the marine economy, marine ecology, and military defense [2,3,4].

Current main instruments for measuring seawater temperature and depth include the Expendable Bathythermograph (XBT) [5], the Conductivity–Temperature–Depth (CTD) profiler [6], and the Argo global ocean real-time observation network [7]. The XBT is widely used due to its ease of deployment, high efficiency, and low hardware costs, making it an effective tool for rapid temperature–depth measurements. However, the traditional XBT does not have a pressure measurement component and has historically relied on empirical formulas based on the probe’s time and displacement to estimate the depth at a given moment. Some research has focused on optimizing the probe’s deployment angle, casing shape, and water-entry posture to improve the accuracy of these empirical formulas [8], but challenges persist in regions with complex ocean currents. To address the lack of a pressure measurement component, some studies have added pressure measurement structures to obtain independent depth data [9,10]. In this case, it is crucial for the temperature and depth data acquisition times to be synchronized. If there is a significant time lag, temperature data may drift relative to depth data, affecting the accuracy of the temperature–depth profile. The most significant limitation of traditional XBTs, however, lies in their use of electrical temperature measurement elements, such as platinum resistance thermometers and thermistors, which exhibit a lower sensitivity, slower response times, and a poor thermal stability [11]. Therefore, replacing traditional XBT electrical temperature sensors with new sensing elements is a key direction for the development of XBT technology.

With the gradual advancement of optical fiber sensing technology, it has found widespread applications in fields such as electric power [12,13,14], petroleum [15], chemical engineering [16], construction [17,18,19], transportation [20], medical care [21], environmental protection, and military applications [22,23,24]. Optical fiber sensors, especially in the precise measurement of physical parameters like temperature, strain, and displacement, have demonstrated unique technical advantages. Currently, research on optical fiber sensors is primarily divided into two main directions: one involves distributed measurements based on backward light scattering or forward light interference, while the other focuses on multi-point measurements using fiber Bragg gratings (FBGs). Based on the characteristics of fiber scattering, optical fiber sensing technologies can be classified into Rayleigh fiber sensing, Brillouin fiber sensing, and Raman fiber sensing. Among these, Brillouin optical fiber sensing can measure both temperature and strain distributions along the fiber, providing a high spatial resolution over long sensing distances. In contrast, Raman optical fiber sensing is used for large-scale distributed temperature monitoring. Unlike Brillouin scattering, the Raman scattering measurement system is not sensitive to strain, which eliminates cross-sensitivity between temperature and strain measurements [25]. Traditional Raman distributed optical fiber sensing requires the significant accumulation and averaging of scattering signals to reduce noise; however, this process increases the measurement time. Additionally, the Raman effect is only sensitive to temperature and cannot effectively demodulate other physical parameters along the fiber, limiting its capacity for multi-parameter detection. For conventional Brillouin-based distributed sensing technologies, temperature and strain measurement times typically range from a few seconds to several minutes, which limits their application in rapidly changing environments [26]. Distributed optical fiber sensors based on Brillouin or Raman technologies are capable of detecting temperature and pressure [27]; though, their relatively long measurement times make them unsuitable for the real-time, fast detection of ocean data using XBT. In this context, multi-point measurement based on fiber Bragg gratings offers a practical solution.

The development of FBG technology is an important branch of fiber optic technology. Compared with traditional electrical sensors like strain gauges, FBGs offer numerous advantages, including their small size [28], high-temperature resistance [29], corrosion resistance [30], electromagnetic interference immunity [31], low loss [32], corrosion resistance [33], electrical insulation, and resistance to electromagnetic interference [34]. As excellent elements for strain and temperature measurement, FBGs have been widely investigated in various fields in recent years. Although FBGs are not yet the mainstream sensing technology in marine applications, they can be favorably used to address issues such as poor thermal stability associated with traditional XBTs, especially given their advantages in temperature and stress measurement [35]. Moreover, FBGs are very cost-effective, which significantly reduces the cost of sea trials for expendable devices like XBTs. The research on applying FBGs in XBTs complements traditional marine electrical sensors and is of great significance for marine engineering applications.

This paper analyzes the research progress in the application of FBGs in ocean temperature and depth sensing, summarizes and compares the advantages and disadvantages of different enhancement techniques, and concludes that the design of independent temperature and pressure measurement structures, along with the synchronization of their dual-parameter acquisition speeds, has become an important research direction in optical fiber sensing technology. These advancements offer new insights and methods for the application of FBGs in deep-sea temperature–depth data acquisition.

## 2. Current Applications of Fiber Bragg Grating-Based Ocean Temperature and Depth Sensors

At present, FBGs are widely utilized in diverse engineering and scientific disciplines. However, due to the low sensitivity of bare FBGs, they are not suitable for high-precision detection in engineering applications. To address this, various sensitivity enhancement methods have been developed to improve the temperature and pressure sensitivity of FBGs. This chapter provides a detailed overview of recent global research progress on FBG-based ocean temperature and pressure sensors. Additionally, comparison and analyses have been given on the advantages and disadvantages of different sensitivity enhancement techniques.

### 2.1. Principle of Fiber Bragg Grating Sensing

In a standard optical fiber, the core is made of uniformly distributed fused silica, with a constant and equal refractive index distribution. By employing methods such as femtosecond laser inscription [36] or ultraviolet (UV) light exposure [37], the refractive index of the quartz material can be locally altered. These methods are used to inscribe several equally spaced, narrow strips with refractive indices differing from the rest of the fiber, forming a grating structure, known as an FBG.

**Figure 1:**
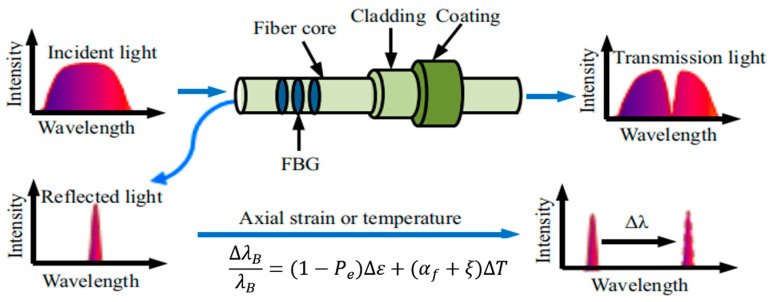
Principle of Fiber Bragg Grating Reflection and Transmission [38].

When a broad-spectrum light is launched into a fiber with an FBG sensor, a portion of its energy will be transmitted through, and another portion will be reflected for a particular wavelength, named the Bragg wavelength *λ_B_*, which satisfies the Bragg condition (*λ_B_ = 2η_eff_Λ*). Then, the principle of sensing using an FBG sensor is that both the effective refraction in dex (*η_eff_)* as well as the periodic spacing between the grating planes (Λ) is affected by external perturbations such as strain and temperature, inducing a shift in the reflected wavelength [38], as shown in Figure 1.

Exposing the fiber with the FBG to a temperature change Δ*T* or stretching/compressing it Δ*l* will spectrally shift Δ*λ_B_* proportionally to the external action. In this case, the relationship between the Bragg wavelength and temperature or strain can be described as follows [39]:

(1) $$\Delta \lambda_{B}=2\left(n_{eff}\frac{d\Lambda }{dT}+\Lambda \frac{dn_{eff}}{dT}\right)\Delta T+2\left(n_{eff}\frac{d\Lambda }{dl}+\Lambda \frac{dn_{eff}}{dl}\right)\Delta l$$

The two parameters influencing the shift in the reflection wavelength of an FBG are the effective refractive index and the grating period. Therefore, by measuring the wavelength shift, the external environmental changes can be quantitatively described. Temperature and strain are the most significant factors causing wavelength shift. When an FBG is affected by the external temperature, both the grating period and the refractive index change due to thermal expansion and the thermo-optic effect. Similarly, under external stress, these two parameters also undergo variations. Thus, measurements of environmental changes are fundamentally based on alterations in temperature or strain. The following sections will discuss these factors individually.

### 2.2. Fiber Bragg Grating Temperature Sensing

#### 2.2.1. Principle of FBG Temperature Sensing

**Figure 2:**
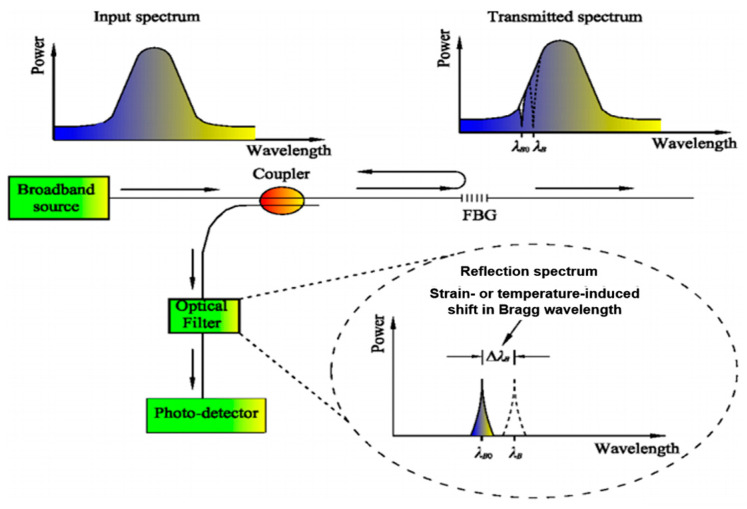
Spectral Shift in Fiber Bragg Grating under External Influences [40].

During light transmission, the grating reflects light at specific frequencies, and the reflected frequency is influenced by temperature [40], as illustrated in Figure 2.

When the external temperature changes, the wavelength of the reflected light shifts accordingly. This process is primarily affected by two factors: the thermo-optic coefficient *(ζ)* and the thermal expansion coefficient (*α*) of the optical fiber. Changes in these parameters alter the effective refractive index of the waveguide and the grating period. Under a temperature variation Δ*T*, the wavelength shift in the FBG can be expressed as follows:

(2) $$\frac{\Delta \lambda_{B}}{\lambda_{B}}=\left(\alpha +\xi \right)\Delta T$$

where *α* is the linear thermal expansion coefficient of the FBG, and *ξ* is the thermo-optic coefficient of the FBG.

#### 2.2.2. FBG Temperature Sensitivity Enhancement Techniques

Bare FBGs exhibit a poor temperature sensitivity, with a typical sensitivity of approximately 0.0082–0.012 nm/°C at a wavelength of 1550 nm [39,41]. To improve their sensitivity, FBG sensors are encapsulated within materials that possess higher thermal expansion coefficients, thereby enhancing the temperature sensitivity. Common encapsulation techniques include metal or polymer coatings [42,43,44,45], fusion coating [46], chemical plating [47], and the tapering of optical fibers [48], all of which have demonstrated effective sensitivity enhancements.

Significant progress has been achieved globally in enhancing FBG temperature sensitivity using coating methods (e.g., metals or polymers). Since 2006, S. Sandlin et al. employed metallic coatings (such as silver and nickel) and found that the temperature sensitivity of metallized FBGs increased significantly, reaching 15 pm/K. This demonstrated that metallic coatings markedly improve the temperature sensitivity of FBG sensors [49,50].

Beyond metallic coatings, B. Wei et al. conducted low-temperature sensing experiments on FBGs coated with acrylic and polyimide. Their results showed that both materials enhanced the temperature sensitivity of FBGs within a range of 55.15 K to 298.15 K. However, the wavelength shifts in these coated FBGs were nonlinear with temperature changes [51]. In 2013, to compare the effects of different metal coatings on the sensitivity of FBGs, Wang et al. applied gold, copper, and zinc coatings to the surface of optical fiber gratings and conducted continuous operation within a thermal test chamber, spanning a temperature range of 0 °C to 95 °C. Multiple experiments demonstrated that the zinc-coated optical fiber grating exhibited the highest temperature sensitivity, reaching 0.0235 nm/°C. However, this sensor is not suitable for operation in the complex environmental conditions typically encountered in marine detection, where factors such as salinity and humidity may interfere with its performance [52]. In 2017, Qu et al. introduced a diamond-like carbon (DLC) thin-film coating on metallized FBGs; their experiments demonstrated a temperature sensitivity of 21.86 pm/°C, with the coating providing corrosion resistance suitable for marine environments [53].

In 2019, Liu et al. from Beijing Jiaotong University used FBG temperature sensors to study high-temperature superconducting materials. They compared the temperature characteristics of bare FBGs with those coated with gold or silver within a range of 77–293 K, revealing significant enhancements in sensitivity for the coated sensors [54]. That same year, Yan et al. encapsulated FBGs with aluminum and conducted experiments within a 10 °C to 60 °C range. Their results showed a temperature sensitivity of 40.4 pm/°C, four times that of bare FBGs [55].

Over the years, extensive research has been conducted on metallic or polymer-encapsulated FBG techniques, yielding numerous advancements. In 2011, Wang Peng et al. proposed a novel packaging method combining polymers with copper capillary tubes. Their study of the temperature sensing characteristics of FBGs encapsulated in copper tubes demonstrated a linearity of 99.929% and sensitivity of 13.675 pm/°C, confirming the feasibility of copper capillary encapsulation [56].

Subsequently, in 2014, Wang Yongjie et al. designed a novel FBG temperature sensor using a tubular dual-end encapsulation structure with a copper material. This sensor achieved a response time of 48.6 ms, meeting the requirements of commercial electrical sensors for certain applications in marine environments. However, its sensitivity remained relatively low. In 2023, Long L. et al. developed a sensitized FBG temperature sensor within a substrate-based encapsulation structure. Using an aluminum alloy with a high thermal expansion coefficient, they conducted simulations and optimizations via ANSYS software. The study revealed that the encapsulated FBG sensor exhibited a sensitivity of 27.3 pm/°C within a range of −20~40 °C, 2.7 times higher than that of bare FBGs, with a linearity exceeding 0.99 [57]. In the same year, Chen et al. designed a structure based on an FBG cascade, in which a droplet-shaped structure was formed by bending a section of coating-stripped fiber at the tail of the FBG. The tilted central wavelength caused by the droplet-shaped structure was influenced by the surrounding refractive index (RI) and temperature, while the tilted central wavelength induced by the FBG was solely related to temperature, thus enabling temperature measurement [58]. Experimental results demonstrated the feasibility of this structure, with a temperature sensitivity of 10.3 pm/°C. Subsequently, in 2018, Arnaldo Leal-Junior et al. conducted experiments embedding FBGs into polylatic acid (PLA) and thermoplastic polyurethane (TPU) structures. The results indicated good linearity for both structures, with response times of less than 2 s. The temperature sensitivity of the FBG embedded in the PLA structure reached 139 pm/°C [59].

With the continuous application of specialty fiber materials in temperature measurement, in 2019, Pan et al. utilized the phase mask method to inscribe a novel FBG in PbS-doped silica fiber, and conducted its performance testing [60]. The experimental results showed that the FBG-PbS exhibited a temperature sensitivity of 19 pm/°C in the range of 20–150 °C, with excellent linearity and repeatability. Compared to the temperature sensitivity of FBGs in standard single-mode fibers (SMF), an improvement of approximately 60% was achieved.

Based on previous research on FBG cascaded structures, Li et al. proposed a structure in 2021 that integrates an MZI with an FBG cascade. Experimental results demonstrated that under conditions of 25 °C to 80 °C, the FBG exhibited a temperature sensitivity of 12.25 pm/°C and enabled the simultaneous acquisition of RI and temperature parameters, showcasing the broad application range of FBGs [61]. However, the temperature sensitivity enhancement of the sensor was relatively insignificant, attributed to the different responses of the MZI and FBG to RI and temperature changes. This configuration effectively addressed the issue of temperature cross-sensitivity in RI sensors. Nevertheless, the sensor remains unsuitable for ocean temperature detection within the range of −5 °C to 35 °C.

As shown in Table 1, using metal coatings or encapsulating FBGs directly with metals demonstrates significantly better sensitivity enhancement compared to using organic material coatings. Moreover, direct encapsulation with metals is much simpler than applying metal coatings. Therefore, directly encapsulating FBGs with metals that have high thermal expansion and thermal conductivity coefficients is an effective approach for enhancing the temperature sensitivity of FBGs. Additionally, given the complex and dynamic nature of the marine environment, sensors used for ocean temperature detection (typically in the range of −5 °C to 35 °C) must also consider performance aspects such as the measurement range, interference resistance, high-pressure tolerance, corrosion resistance, and waterproofing capabilities.

### 2.3. Fiber Bragg Grating Pressure Sensing

The external pressure-induced deformation of FBG can be categorized into axial deformation and bending deformation. The principles of axial strain and bending strain will be introduced separately below.

#### 2.3.1. Principle of Axial Strain Sensing with Fiber Bragg Gratings

When uniform axial pressure is applied to the FBG, an axial strain (*ε*) is generated, leading to a change in its grating period. The axial strain ε can be expressed as

(3) $$\varepsilon =\frac{\Delta \Lambda }{\Lambda }=\frac{\Delta x}{x}$$

where *∆x* is the deformation of the FBG, x is the effective length of the FBG, and the strain in the FBG also changes its effective refractive index, which can be expressed as

(4) $$\frac{\Delta n_{eff}}{n_{eff}}=-\frac{1}{2}{n_{eff}}^{2}\left[p_{12}-\mu \left(p_{12}+p_{11}\right)\right]\cdot \varepsilon$$

In the equation, *p*_11_ and *p*_12_ are the photoelastic coefficients of the optical fiber, and *μ* is the Poisson’s ratio of the fiber material. The effective photoelastic coefficient is then given by

(5) $$P_{e}=-\frac{1}{2}{n_{eff}}^{2}\left[p_{12}-\mu \left(p_{11}+p_{12}\right)\right]$$

By combining the above equations, the wavelength shift caused by the axial strain can be expressed as

(6) $$\Delta \lambda_{b}=\left(1-P_{e}\right)\lambda \cdot \varepsilon$$

For the FBG with a fixed central wavelength and material, its wavelength shift is solely dependent on the strain applied to the FBG. Therefore, FBG can be used for pressure sensing.

#### 2.3.2. Fiber Bragg Grating Bending Strain Sensing Principle

Fiber bending causes a displacement of the fundamental mode field, which increases the dielectric constant and consequently affects the refractive index of the fiber. To solve for the distribution of the effective refractive index of the fiber under bending, the uniformly bent fiber is equivalently treated as a non-uniform straight fiber to calculate the effective refractive index, *n_eff_.*

The ideal wave equation for the bent fiber is as follows:

(7) $$\Delta \lambda_{B}=\lambda_{B}(1-P)\cdot \varepsilon$$

(8) $$\varepsilon =\frac{h}{2R}$$

In the equation,

$\Delta \lambda_{B}$—wavelength change;

$\lambda_{B}$—original Bragg wavelength;

*P*—optical Poisson’s ratio of the optical fiber material;

ε—strain;

*h*—diameter of the optical fiber;

*R*—bending radius.

From this formula, it can be seen that when the FBG is bent, the change in its curvature directly affects the wavelength shift. Generally speaking, the greater the curvature, the larger the wavelength shift. This is because when the fiber is bent, the propagation path of the light within the fiber changes, leading to a modification in the refractive index modulation period of the grating, which in turn causes the center wavelength to shift.

#### 2.3.3. Fiber Bragg Grating Pressure Sensitization Techniques

Due to the low intrinsic pressure sensitivity of bare FBGs (only 8 pm/MPa) [63], enhancing the sensitivity to strain or pressure is primarily achieved by amplifying the stress or deformation exerted on the FBG. Various techniques have been developed for the pressure sensitization of FBGs, including elastic diaphragms [64], polymers [65], thin-walled cylinders [66], and corrugated tubes [67]. These techniques can be broadly categorized into four groups: polymer-based sensitization, corrugated tube-based sensitization, thin-walled cylinder-based sensitization, and elastic diaphragm-based sensitization, as detailed below.

##### Polymer-Based Sensitization

Polymer-based sensitization involves embedding the FBG into a polymer material. Pressure P is applied axially or radially to the polymer surface, which acts as a sensor and transmits the strain to the FBG [68]. Additionally, the polymer layer provides protection for the FBG, shielding it from external environmental factors that could lead to damage. This protective feature extends the lifespan and stability of the FBG, as it is inherently fragile and prone to environmental disturbances.

In 2014, K. Bhowmik et al. investigated the intrinsic hydrostatic pressure sensitivity of polymer optical fiber Bragg gratings (POFBGs) with different diameters. The POFBGs were inscribed into single-mode polymer fibers and etched to various diameters. Under hydrostatic pressure ranging from 0 to 1 MPa, the experimental results revealed that a POFBG with a diameter of 55 μm exhibited a pressure sensitivity of 0.75 pm/kPa. However, as this experiment was conducted under static hydrostatic pressure conditions and within a limited range (0–1 MPa), it is unsuitable for the dynamic and complex environments encountered in marine applications [69]. In 2018, Boxin Mu and colleagues from Harbin Engineering University encapsulated FBGs within rubber and compared two methods of deployment: inserting the encapsulated FBG into a bearing and embedding it on the bearing surface. The experiments demonstrated that the insertion method provided a higher pressure sensitivity, achieving 0.38 nm/MPa within the range of 0–0.1 MPa [70]. This structure significantly enhanced the pressure sensitivity in low-pressure and small-scale applications, making it suitable for high-precision measurements in low-pressure environments. Building on this foundation, in 2023, Wenjie Dang and colleagues from Southeast University utilized high-temperature adhesive (HTA) to encapsulate and seal FBG sensors with different cladding diameters into hollow silica tubes (HCSTs). These sensors were used to measure the gas pressure under high-temperature conditions. Experimental results demonstrated that the sensors achieved a maximum pressure sensitivity of 0.993 nm/MPa within a temperature–pressure range of 17–400 °C and 0–1 MPa [71]. Compared to earlier designs, this sensor exhibited a significantly improved sensitivity and an expanded detection range, making it suitable for marine environments, such as detecting pressures at depths of up to 100 m underwater (approximated as 1 MPa).

In 2024, C.L. Abeywardena et al. proposed a highly sensitive contact-based FBG pressure sensor, simulating the effects of different polymer materials with varying Young’s moduli. According to the simulation results, by embedding a 3 mm long FBG at the horizontal center of a polymer layer with a Young’s modulus of 20 MPa, a disc-shaped structure with a diameter of 5.5 mm and a thickness of 1 mm was formed. This design enhanced the pressure sensitivity of the bare FBG to 0.8179 nm/MPa [65]. The proposed sensor structure not only achieved a high sensitivity, but also significantly expanded the detection range.

##### Bellows-Based Sensitivity Enhancement

Polymer-based sensitivity enhancement may suffer from creep deformation and aging, which can severely limit the durability of sensors. Bellows-based sensitivity enhancement, however, effectively avoids these issues. A bellows is a tubular elastic sensing element constructed by connecting collapsible corrugated sheets along the direction of expansion and contraction. Typically, bellows have thin walls, making them prone to elastic deformation under external pressure or force. By embedding or attaching an FBG onto the bellows, the elastic deformation of the bellows under external pressure is transferred to the FBG, inducing strain in the grating.

In 2009, Song et al. proposed an optical pressure sensor based on an FBG and metallic bellows. Due to the low spring rate of the metallic bellows, the sensitivity of the FBG was increased to 48 pm/kPa compared to bare FBGs. The experimental results demonstrated a strong linear relationship between the Bragg wavelength shift and the applied pressure, confirming the sensitivity enhancement achieved by encapsulating the FBG with the metallic bellows [72].

**Figure 3:**
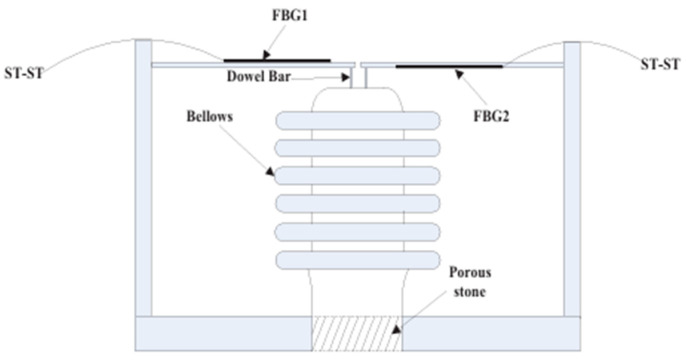
Schematic Diagram of Pressure Sensor Structure [74].

In 2013, Yang et al. designed a pressure sensor utilizing dual bellows for the encapsulation of a metallized FBG. An experimental system for the dual-bellows FBG pressure sensor was established, revealing a pressure sensitivity coefficient of 184.98 nm/MPa within a measurement range of 0–10 kPa. The correlation coefficient exceeded 99.9%, with a resolution of up to 0.05% [73]. While the dual-bellows structure demonstrated high sensitivity, it was limited in measurement range. In 2017, Chen et al. developed a differential pressure sensor combining bellows, a triangular cantilever beam, and an FBG. Experiments conducted in the 100–200 kPa range demonstrated a pressure sensitivity of 1056 pm/kPa with an excellent linearity [74], as shown in Figure 3.

Additionally, this sensor incorporated temperature compensation. Although its measurement range was limited, modifying the bellows design and adjusting cantilever beam parameters could extend its range, marking a notable improvement over previous designs. With further research into bellows-encapsulated structures, Wang et al. introduced a carbon coating and bellows-filling technique in 2021. This design achieved efficient pressure sensing through bellows encapsulation while protecting the FBG from corrosion [75]. The experimental results indicated that the FBG exhibited an excellent pressure linearity, making it suitable for dynamic monitoring in high-pressure environments. This structure provides a valuable reference for designing pressure sensors for oceanographic applications.

##### Sensitivity Enhancement Using Thin-Walled Cylinders

The principle of sensitivity enhancement for FBGs using thin-walled cylinders involves combining the mechanical characteristics of the cylinder with the sensitivity of the FBG. Typically, the FBG is attached to the outer surface of the thin-walled cylinder, either in the axial or circumferential direction. When the cylinder is subjected to external pressure, the resulting strain is transferred to the FBG, causing it to undergo tensile or compressive deformation.

In 2016, Gu et al. proposed a practical fiber optic sensor for hydraulic pressure measurement using a thin-walled cylindrical structure. Experiments conducted in the range of 0–16 MPa demonstrated a pressure sensitivity of 69.4 pm/MPa [66]. The sensor’s advantages include small size, ease of fabrication, structural stability, and a wide measurement range. Moreover, its performance can be tailored by altering material and structural parameters, making it a valuable reference for designing oceanographic pressure sensors. However, its pressure sensitivity falls short of the high demands for marine environmental detection, necessitating further improvements.

In 2018, Zhao et al. mounted a pair of FBGs on the outer wall of a thin-walled cylinder for pressure measurement. The resulting pressure sensitivity was 0.15414 nm/MPa, with a linearity exceeding 99.99% [76]. This design demonstrated an improved sensitivity and linearity compared to its predecessor. In 2022, Van Quyet Nguyen et al. from National Kaohsiung University of Science and Technology utilized 3D printing to fabricate an elliptical cylinder structure. Deformations in the elliptical cylinder transferred the strain to the FBG, resulting in a pressure sensitivity of 6.834 nm/MPa within a pressure range of 0–0.45 MPa [77]. While this sensor achieved a significant increase in sensitivity compared to earlier designs, its limited detection range rendered it unsuitable for deep-sea applications. However, it holds promise for low-pressure environments requiring high-precision detection.

**Figure 4:**
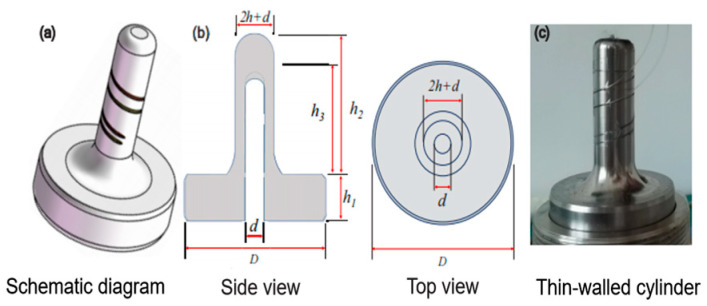
(**a**–**c**) Principle and Physical Diagram of π-FBG Sensor [67].

To address the limitations of many sensors in deep-sea high-pressure environments, in 2023, Ji et al. reported a high-resolution, wide-range pressure sensor based on a thin-walled metal cylinder encapsulating a π-phase-shifted FBG (π-FBG). The sensor, shown in Figure 4, was calibrated for temperature and tested in the range of 0–110 MPa, achieving a sensitivity of 4.42 pm/MPa and excellent repeatability [67]. Despite its lower sensitivity compared to previous designs, the simple structure, excellent repeatability, and high practicality of this sensor make it well-suited for deep-sea or high-pressure applications.

##### Sensitivity Enhancement Using Elastic Diaphragms

The use of diaphragms to enhance the sensitivity of FBG pressure sensors has become a popular method in recent years. The effectiveness of this approach can vary depending on the material and shape of the diaphragm. The key advantages of diaphragm-based sensitivity enhancement include its ease of operation and the ability to create high-sensitivity or wide-range sensors by cascading multiple diaphragms. Generally, diaphragm-based enhancement can be categorized into two types: the first involves using the axial strain of the diaphragm under pressure to produce displacement on the FBG, while the second uses the diaphragm’s bending strain, directly transferring it to the FBG. Elastic diaphragm-based sensitivity enhancement can be further classified into the polymer diaphragm and metal diaphragm.

Polymer diaphragms not only enhance sensitivity but also provide an additional layer of protection to the FBG, increasing its mechanical strength and reducing the risk of damage from external forces. For example, in 2019, Leal-Junior A. G. et al. developed an FBG sensor based on a polymer diaphragm, which involved embedding the FBG in a PTFE Polytetrafluoroethylene capillary tube. The experiment, conducted at a constant temperature of 28 °C, with a pressure range of 0–80 kPa, showed that the temperature and pressure errors were about 6% [78]. However, since the structure only included one FBG for dual-parameter testing (temperature and pressure), it was susceptible to random errors that could affect the sensor accuracy. Moreover, the sensor’s sensitivity, linearity, and dynamic range need further enhancement.

In recent years, the use of metal diaphragms for FBG sensitivity enhancement has become more common. Metal diaphragms offer an excellent corrosion resistance, high-temperature tolerance, and wear resistance, improving the stability of FBGs in harsh environments. Additionally, metal diaphragms provide electromagnetic shielding, making these sensors suitable for complex electromagnetic environments. For instance, in 2015, Allwood et al. described a pressure sensor based on an FBG mounted on a flat circular rubber diaphragm adhered to a cylindrical body. The sensor exhibited a sensitivity of 0.116 nm/kPa within a measurement range of 0–15 kPa [79]. Although the sensor showed a good sensitivity, it lacked temperature compensation, which could lead to temperature-related errors. Moreover, the limited measurement range makes it suitable only for small-scale, high-precision detection. In 2018, Liang et al. proposed an FBG pressure sensor based on a circular flat diaphragm, incorporating temperature compensation via a temperature-compensated FBG. This sensor achieved a pressure sensitivity of 35.7 pm/MPa in the 0–50 MPa range, with a linearity of 99.95% [80]. The simple design, along with temperature compensation, provided a high sensitivity and linearity, making it suitable for oceanic pressure measurement.

Common pressure sensors use various methods for temperature compensation to reduce the impact of temperature on their performance, but most of these methods are quite complex. To address this issue, in 2019, V.V.S. Ch.Swamy et al. introduced a low-cost, diaphragm-based FBG pressure sensor. The sensor featured a single bare FBG mounted at the center of a circular diaphragm, enabling maximum pressure application at the diaphragm’s center. This design was effective for both static water pressure measurement and dynamic marine pressure detection, although its measurement range was limited to 0–0.5 MPa, making it suitable for shallow marine environments [81].

In 2021, Hedge et al. developed a temperature-compensated FBG-based high-pressure sensor with a diaphragm, capable of measuring pressures up to 700 bar. Using a reference grating for temperature compensation, the sensor could measure pressures with high precision in the temperature range of −40 °C to 90 °C, with a pressure sensitivity of 36.4 pm/MPa [82]. This sensor’s wide temperature range and good sensitivity make it suitable for high-pressure applications, such as rocket and deep-sea measurements. In addition to the circular diaphragm used in the previous sensor, a sensor combining a square diaphragm with FBG can also achieve pressure sensitivity enhancement for the FBG. In 2021, Fan and others designed an FBG pressure sensor based on a square flat diaphragm. Theoretical and experimental results showed that its pressure sensitivity could reach 3.402 pm/kPa (0~200 kPa). The pressure sensor with a square diaphragm as the sensitivity element exhibits a higher sensitivity than one using a circular diaphragm [83]. This sensor, using a square diaphragm for enhancement, improves the diaphragm’s sensitivity effect to some extent. However, the measurement range of this sensor is not suitable for deep-sea applications.

**Figure 5:**
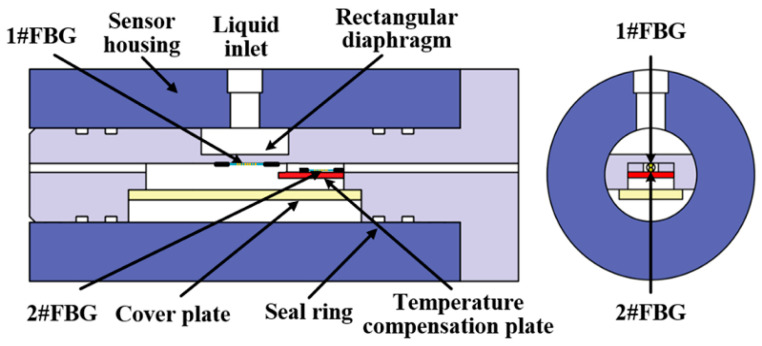
Schematic Diagram of the Structure of an FBG Pressure Sensor [84].

In 2023, Liu Mingyao et al. introduced a novel high-pressure FBG sensor using a regionally homogeneous diaphragm [84], shown in Figure 5. To prevent strain non-uniformity in the FBG bonding zone, they established an optimized model for a regionally homogeneous rectangular diaphragm. The pressure measurement range for this sensor was 0–30 MPa, a significant improvement compared to previous designs. The sensor exhibited a pressure sensitivity of 30.8714 pm/MPa with a linear error of 0.9996 and stable spectral characteristics without chirping. The full width at half maximum (FWHM) fluctuation was only 0.01 nm, demonstrating uniform strain distribution in the FBG bonding area.

##### Comparative Analysis

As shown in Table 2, some studies demonstrate sensitivities several times higher than that of bare FBGs; however, these structures are relatively complex and do not account for the temperature compensation and the response time of the optical fiber. The use of elastic diaphragms for sensitivity enhancement is simple, compact, easy to process, and allows for the flexible design of the required sensor’s measurement range and sensitivity by adjusting the diaphragm’s diameter, thickness, and material. The thin-walled cylinder method provides good pressure sensitivity enhancement, and mostly, with pressure sensitivities improved by at least one order of magnitude compared to bare FBGs. However, this method is more complex and requires more technical processes and labor, which significantly increases costs. Additionally, to be suitable for marine pressure sensing, its seawater corrosion resistance needs to be considered.

### 2.4. Fiber Bragg Grating Temperature and Pressure Sensing

#### 2.4.1. Principles of Fiber Bragg Grating Temperature and Pressure Sensing

The peak wavelength of a fiber Bragg grating is expressed as

(9) $$\lambda_{B}=2n_{eff}\Lambda$$

where n_eff_ is the effective refractive index of the fiber core, and *Λ* is the grating period. When either the refractive index of the core or the grating period changes, the shift in the Bragg wavelength is given by

(10) $${\Delta \lambda }_{B}=2\Lambda {\Delta n}_{eff}+2n_{eff}\Delta \Lambda$$

When both temperature and strain vary simultaneously, the corresponding Bragg wavelength shift is

(11) $${\Delta \lambda }_{B}=2n_{eff}\Lambda \left[\left\{1-\left(\frac{n^{2}}{2}\right)\left[p_{12}-v\left(P_{11}+p_{12}\right)\right]\right\}\Delta \varepsilon +\left[\alpha +\xi \right]\Delta T\right]$$

where Δε is the strain variation, ΔT is the temperature variation, P_ij_ is the strain-optic coefficient of the fiber material, *α* is the thermal expansion coefficient of the fiber material, and *ζ* is the thermo-optic coefficient. v is the Poisson’s ratio of the optical fiber material. The wavelength responses for the strain variation at a constant temperature and the temperature variation at a constant strain (with the operating wavelength denoted as *λ_B_* = 1550 nm) are expressed as follows:

(12) $$\frac{{\Delta \lambda }_{B\epsilon }}{\Delta \varepsilon }=1.20\times 10^{-3}\;nm/\mu \varepsilon$$

where ${\Delta \lambda }_{B\epsilon }$ represents the wavelength change induced by the strain.

(13) $$\frac{{\Delta \lambda }_{BT}}{\Delta \varepsilon }=10.3\times 10^{-3}\;nm/{}^{\circ}C$$

where ${\Delta \lambda }_{BT}$ represents the wavelength change induced by the temperature.

#### 2.4.2. Temperature and Pressure Sensors Based on Fiber Bragg Grating

In 2014, Duraibabu and his team developed a sensor combining an extrinsic fiber Fabry–Pérot interferometer (EFPI) with an FBG to achieve the real-time monitoring of marine environments [85]. This sensor replaced conventional electrical temperature-sensing elements with FBG as the sensing element, improving the stability of the detection process. However, the sensor did not demonstrate significant advantages in the sensitivity or response speed. With the progressive advancement of Fabry–Pérot interferometric techniques, in 2020, K. M. Fadeev et al. proposed a sensor integrating a Fabry–Pérot interferometer and FBG structure for the simultaneous measurement of pressure and temperature [86]. The pressure sensitivity of the sensor was reported as 50 nm/bar. For the temperature measurement, the Fabry–Pérot interferometer side exhibited a sensitivity of 4.6 nm/°, while the FBG side demonstrated a sensitivity of 10 pm/°C. This indicates the feasibility of using FBG as a thermal compensation element.

Although the combination of the Fabry–Pérot interferometer and FBG allows for simultaneous temperature and pressure measurements, the sensitivity advantage is not significant. In addition to this structure, other designs can also achieve dual-parameter sensing. In 2017, Chen et al. installed optical fiber grating temperature and pressure sensors on the pantograph of a railway overhead line to investigate the relationship between these parameters and the current flowing through the pantograph [14]. This sensor separates temperature and pressure measurements, making them independent of each other, which minimizes errors in temperature and depth data. However, it is more suitable for static sensing applications such as pantographs and is not ideal for dynamic sensing applications in marine environments. To design a temperature–depth sensor structure suitable for dynamic and complex marine environments, in 2020, Leal-Junior A. et al. in Brazil encapsulated two gratings in the upper and lower layers of a polyurethane resin diaphragm to measure seawater temperature and depth. Both gratings are sensitive to temperature and depth, with temperature sensitivities of 25.6 pm/°C and 20.3 pm/°C in the range of 20–50 °C, and pressure sensitivities of 0.2 nm/kPa and 0.37 nm/kPa in the depth range of 1.2 kPa [9]. Since both fiber gratings are sensitive to temperature and depth, this complicates further data processing, and its pressure measurement range is relatively small, making it more suitable for shallow marine applications.

In 2018, Liu et al. proposed a novel optical fiber combined sensor for acoustics, temperature, and pressure measurements, which connects an FBG and a Michelson interferometric optical fiber hydrophone to independently measure and perform temperature compensation for the pressure sensor part. Experiments conducted at 5–45 °C and 0–8 MPa showed a temperature sensitivity of 9.4 pm/°C and pressure sensitivity of 105.9 pm/MPa [87]. This sensor achieved dynamic water detection with a wider detection range than previous studies, but it is still more suited to shallow marine detection and has relatively low temperature sensitivity. In 2021, Wang X et al. proposed an optical fiber dual-cavity FP interferometric sensor for simultaneous temperature and pressure measurement. The sensor cascades an intrinsic FP interferometer and a non-intrinsic FP interferometer. The intrinsic FP interferometer serves as a temperature sensor, while the non-intrinsic FP interferometer acts as a pressure sensor. It exhibits a temperature sensitivity of 29.63 nm/°C in the 40–1100 °C range and a pressure sensitivity of 1465.8 nm/MPa in the 0–10 MPa pressure range [10]. This sensor demonstrates a high temperature and pressure sensitivity, suitable for dynamic environmental detection, with a wide pressure range. However, because temperature and pressure are measured independently, the mismatch in the dual-parameter acquisition time leads to a reduction in the data accuracy when applied to marine environments.

By comparing the various temperature–depth sensor structures discussed above, we find that marine temperature–depth sensors require a temperature sensing range of −5 °C to 35 °C. However, several sensors with an enhanced sensitivity have a minimum detection temperature above 20 °C. For example, the FBG-based temperature–depth sensor encapsulated in a polyurethane resin membrane has a temperature detection range of 20 °C to 50 °C, while the FBG dual-cavity FP interferometric sensor, used for simultaneous temperature and pressure measurements, has a temperature sensing range of 40 °C to 1100 °C. Such sensors are not suitable for marine applications. When the temperature and depth are measured independently, temperature compensation structures can be used to eliminate temperature errors in pressure sensing fiber gratings. However, when these sensors independently measure temperature and pressure, the mismatch in the acquisition time for the two parameters also reduces the sensor’s accuracy. Therefore, marine temperature–depth sensors must not only have a high sensitivity and response speed for the temperature and pressure, but also address issues such as the detection range, dual-parameter acquisition speed matching, and temperature compensation. Although these issues do not directly affect the sensor sensitivity, they can introduce non-negligible errors in the final accuracy.

**Figure 6:**
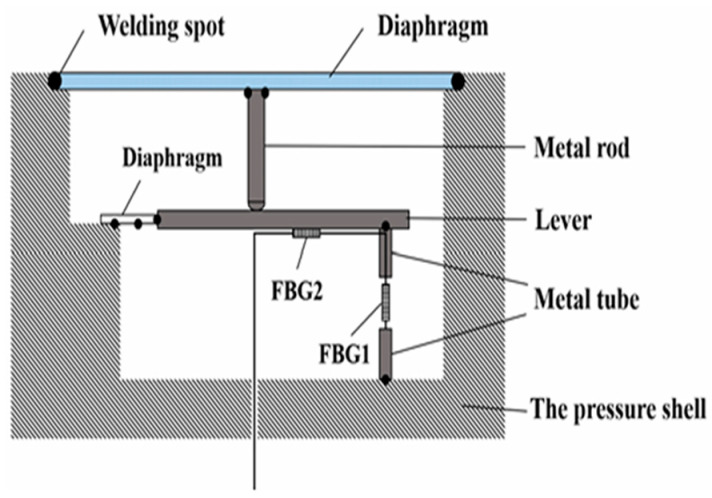
Schematic Diagram of the Sensor Structure Based on Membrane and Lever [86].

To address this issue, in 2022, Liu Zhaoyue and other project team members proposed a temperature–depth sensing structure based on a diaphragm and liquid filling, using thermal expansion and diaphragm deformation to enhance the sensitivity of both FBGs for temperature and depth measurements. In the 0–8 MPa range, the temperature sensitivity of the sensor is 1.065 nm/°C, and the pressure sensitivity is 1.245 nm/MPa, with a response time of approximately 51 ms [88]. This sensor improved the sensitivity of temperature and depth measurements compared to previous temperature–depth sensors and solved the mismatch in the dual-parameter acquisition time, although it has a slower response time. In the same year, Liu Zhaoyue and others further proposed a temperature–depth sensing structure based on a diaphragm and lever mechanism, as shown in Figure 6. Simulations showed that this structure, using the lever mechanism for the secondary sensitivity enhancement of the diaphragm, improves the pressure sensitivity compared to a single diaphragm structure. In the 0–8 MPa range, the temperature sensitivity is 31.7 pm/°C and the pressure sensitivity is 4.21 nm/MPa [89]. The response time of both the temperature and depth is less than 30 ms, improving both the response speed and the resolution of the timing mismatch issue.

Although these two structures address the issues of a slow response speed, low sensitivity, and dual-parameter acquisition time mismatch, they are designed for a pressure range of 0–8 MPa (corresponding to a 0–800 m depth in the ocean). They are suited for shallow marine exploration. However, due to their enhanced sensitivity, the methods used in these structures do not protect the fiber gratings. When applied to deep-sea high-pressure environments, the fiber gratings will be subjected to pressures far exceeding their maximum capacity, causing the gratings to fail before they can complete the measurement.

To resolve this problem, the research team continued their studies and finally proposed a marine temperature–depth sensor based on diaphragm and bending strain. This sensor uses a rectangular copper diaphragm to directly transmit strain to the fiber grating for the pressure measurement and a metal silver tube to transmit temperature directly to the fiber grating for the temperature measurement using thermal expansion. This structure realizes independent temperature and depth measurements. The experimental analysis showed that this structure, within the 0–20 MPa range, has a temperature and depth response time of less than 15 ms, with a pressure sensitivity greater than 50 pm/MPa and temperature sensitivity greater than 35 pm/°C. This structure not only increases the detection range but also addresses the issues of a slow response speed and dual-parameter acquisition time mismatch. This team is continuing their marine exploration research to meet the detection needs of various marine regions and depths.

## 3. Conclusions

As the core sensing element in marine temperature–depth detection, optical fiber gratings have been optimized through various structural enhancements to meet the diverse requirements of temperature–depth detection in different marine environments and depths. In the design of marine temperature–depth detection enhancement structures using optical fiber gratings, it is essential to not only consider the individual enhancement aspects of temperature and pressure (such as the sensitivity, response speed, and measurement range) but also to examine how the two components may interact with each other. This includes evaluating potential issues, such as whether discrepancies in response speed could lead to temperature data drift in relation to depth, among other concerns. Once these factors are carefully considered, the overall performance of the marine temperature–depth sensor can be comprehensively improved. Moving forward, we will continue our research in marine exploration, designing and optimizing detection structures for different marine regions and depths to develop high-precision marine temperature–depth sensors based on optical fiber gratings as the core sensing element. In addition, salinity and other relevant parameters are crucial in ocean exploration. Recent studies on fiber Bragg gratings have demonstrated that multi-parameter ocean data detection can be achieved through the use of advanced sensor designs with multiple structural configurations [90]. With the advancement of research on fiber Bragg gratings, they have found widespread applications not only in industries such as energy (e.g., thermal effect monitoring and hydrogen concentration monitoring), power industries, oil and gas, and biomedical fields, but are also increasingly being applied in the Internet of Things (IoT), which enables the networking of multiple FBG sensors, creating a scalable and distributed sensing system [91]. This suggests that, in future developments, integrating FBG sensors into large-scale data analysis frameworks for big data applications through the development and optimization of signal processing algorithms is feasible [92]. Moreover, attention should be paid to the miniaturization and simplification of the manufacturing process to enable large-scale production.

## Figures and Tables

**Table 1:** Temperature Sensitization Methods Based on Fiber Bragg Gratings.

Year	Method	Temperature Sensitivity	Temperature Range	Temperature Sensitivity Enhancement	Advantages	Reference
2011	The encapsulation of FBG by combining polymer with capillary copper tubing	13.675 pm/°C	25~110 °C	Copper capillary tube encapsulated FBG	High linearity	[56]
2013	Coating the surface of FBG with gold, copper, and zinc	0.0235 nm/°C	0~95 °C	Coating with materials having a high thermal expansion coefficient	Other	[52]
2015	Acrylic and polyimide coatings on FBG	/	55.15~298.15 K	Using coating materials with high thermal expansion coefficients	Capable of measuring low temperatures	[55]
2015	Integrating FBG with a Fabry–Pérot interferometer using diaphragm shrinkage technology	10.7 pm/°C	/	FBG cascaded with FPI	Compact size	[62]
2015	A structure based on FBG cascading	10.3 pm/°C	20~100 °C	Tail-bent fiber variation	Capable of simultaneously measuring temperature and refractive index (RI)	[59]
2017	Copper electroplating on FBG followed by diamond-like carbon (DLC) thin-film encapsulation	21.86 pm/°C	/	Metal electroplating on FBG	Corrosion-resistant and suitable for marine exploration environments	[53]
2018	Embedding FBG with PLA and TPU	139 pm/°C	20~70 °C	Thermal expansion of polymers	Response time less than 2 s	[60]
2019	Using FBG temperature sensors to study the temperature measurement of high-temperature superconducting materials	/	77~293 K	Coating with metals that have a high thermal expansion coefficient	Other	[54]
2019	Using 7075-T6 aluminum as the substrate to encapsulate the FBG	40.4 pm/°C	10~60 °C	Thermal expansion and contraction of aluminum	The maximum temperature difference does not exceed ±1 °C	[55]
2019	Using the phase mask method to write FBGs on silica fibers doped with PbS	19 pm/°C	20~150 °C	Selecting materials with high thermo-optic coefficients and thermal expansion coefficients	High linearity	[61]
2021	MZI-cascaded FBG-based system	12.25 pm/°C	25~80 °C	MZI-cascaded FBG	Achieving dual-parameter sensing of refractive index (RI) and temperature	[62]
2023	Sensitized FBG temperature sensor in a substrate-type encapsulation structure	27.3 pm/°C	−20~40 °C	Using aluminum alloy with a high thermal expansion coefficient	High linearity	[57]

**Table 2:** Pressure Sensitivity Enhancement Methods Based on FBGs.

Year	Method	Common Features	Pressure Sensitivity	Pressure Range	Pressure Sensitivity Enhancement	Advantages	Reference
2014	Etching of polymer FBGs with different diameters	Changing FBG parameters	0.75 pm/KPa	0~1 MPa	Etching to reduce fiber diameter	Other	[69]
2018	Rubber insertion method for FBG packaging	FBG embedded in a polymer with low Young’s modulus	0.38 nm/MPa	0~0.1 MPa	Rubber shrinkage deformation	Compact size	[70]
2023	Encapsulation of FBG using HTA sealed in a thin hollow quartz tube		0.993 nm/MPa	0~1 MPa	HCST compression deformation	Enhanced sensitivity due to material properties	[71]
2024	High-sensitivity FBG contact pressure sensor		0.8179 nm/ MPa	0~20 MPa	Utilizing low-elastic-modulus materials	Suitable for deep-sea exploration	[65]
2009	FBG combined with metal bellows for fiber optic pressure sensors	FBG embedded/attached to the bellows	48 pm/kPa	/	Utilizing materials with low elastic modulus	Achieving a resolution of 0.05%	[72]
2013	Double bellows encapsulation of metallized FBG		184.98 nm/ MPa	0~10 kPa	Metal welding connections	High sensitivity	[73]
2017	A differential pressure sensor based on the interaction of bellows, a triangular cantilever beam, and FBG		1056 pm/kPa	100~200 kPa	The triangular cantilever beam induces deflection changes	Temperature compensation was implemented	[74]
2021	Carbon coating and corrugated tube packaging FBG		/	0~0.1 MPa	Deformation of the corrugated tube	Dynamic monitoring in high-pressure environments	[75]
2016	Use of thin-walled cylindrical body	Thin-walled cylindrical packaging of FBG for strain transmission	69.4 pm/MPa	0~16 MPa	Deformation through the thin-walled cylinder	Small in volume, easy to fabricate	[66]
2018	A pair of FBGs fixed on the outer wall of a thin-walled cylindrical structure		0.15414 nm/ MPa	/	The cantilever beam undergoes deformation	High linearity	[76]
2022	Deformation transfer to the FBG through the deformation of the elliptical cylinder		6.834 nm/MPa	0~0.45 MPa	Deformation transmitted through the thin-walled cylinder	High sensitivity	[77]
2023	FBG encapsulated in a metal thin-walled cylindrical structure with π phase shift		4.42 pm/MPa	0~110 MPa	Axial expansion deformation of the sidewall	Suitable for deep-sea detection	[67]
2015	FBG is adhered to the diameter of a circular diaphragm	The FBG is embedded/attached to the surface of a circular/ square diaphragm	0.116 nm/kPa	0~15 KPa	The sensitivity is enhanced by using a rubber diaphragm	Suitable for use in harsh environments	[79]
2018	The temperature-compensated pressure sensor based on a circular flat diaphragm		35.7 pm/ MPa	0~50 MPa	The diaphragm undergoes deflection deformation	The structure is simple	[80]
2019	The photosensitive single-mode fiber is embedded in a PTFE capillary tube		/	0~80 kPa	The diaphragm will undergo deflection deformation	The pressure error is small, approximately 6%	[78]
2019	The bare FBG is mounted at the top of the circular diaphragm		24.95 nm/ Mpa; 16.22 nm/ MPa	0~0.5 MPa	The diaphragm undergoes lateral deformation	Suitable for measuring dynamic water pressure	[81]
2021	The FBG is bonded to the center of the circular diaphragm surface		36.4 pm/MPa	Capable of measuring pressures up to 700 bar	The circular diaphragm undergoes deformation	Temperature compensation has been implemented	[82]
2021	The FBG is bonded to the surface of a square flat diaphragm		3.402 pm/kPa	0~200 kPa	The deflection at the center of the square diaphragm causes deformation	The sensitivity enhancement is significantly improved compared to the circular diaphragm	[83]
2023	An optimized model for a regionally homogeneous strain rectangular diaphragm is established		30.8714 pm/MPa	0–30 MPa	Strain of the rectangular diaphragm	Capable of measuring under high-pressure conditions	[84]

## Data Availability

Data are contained within the article.
